# Biology of Human Malaria Plasmodia Including *Plasmodium Knowlesi*

**DOI:** 10.4084/MJHID.2012.013

**Published:** 2012-03-10

**Authors:** Spinello Antinori, Laura Galimberti, Laura Milazzo, Mario Corbellino

**Affiliations:** Department of Clinical Sciences L. Sacco, Section of Infectious Diseases and Immunopathology, University of Milano, Italy

## Abstract

Malaria is a vector-borne infection caused by unicellular parasite of the genus *Plasmodium. Plasmodia* are obligate intracellular parasites that are able to infect and replicate within the erythrocytes after a clinically silent replication phase in the liver. Four species (*P.falciparum*, *P.malariae*, *P.ovale* and *P.vivax*) are traditionally recognized as responsible of natural infection in human beings but the recent upsurge of *P.knowlesi* malaria in South-East Asia has led clinicians to consider it as the fifth human malaria parasite. Recent studies in wild-living apes in Africa have revealed that *P.falciparum*, the most deadly form of human malaria, is not only human-host restricted as previously believed and its phylogenetic lineage is much more complex with new species identified in gorilla, bonobo and chimpanzee. Although less impressive, new data on biology of *P.malariae*, *P.ovale* and *P.vivax* are also emerging and will be briefly discussed in this review.

## Introduction

Four host-restricted or adapted species of *Plasmodium* are traditionally recognized as responsible of human malaria: *Plasmodium falciparum*, *P.malariae*, *P.ovale* and *P.vivax*.^1^ Occasionally, human beings can be infected either naturally or accidentally by several simian species such as *P. cynomolgi cynomolgi*, *P.cynomolgi bastianelli*, *P.simiovale*, *P.brasilianum*, *P.schwetzi*, *P.inui* and *P.knowlesi*.^1^ The latter emerged as an important cause of human malaria in South-East Asia, especially the Malaysian Borneo since 2004 and will be discussed together with the other human plasmodia.^2^

*Plasmodia* have been regarded as belonging to the phylum *Apicomplexa* with an evolution from the Coccidian stem that encompass the progressive acquisition of more complicated phases in their life-cycles confined to a single host. Finally, the successful survival of these parasites requires completion of life cycle in two alternative hosts of evolutionarily distant species (*i.e.* human and mosquito). The genus *Plasmodium* is subdivided in the sub-genus *Plasmodium* and *Laverania* within the sub-order *Haemosporidiidea* **(**Table 1).

*Plasmodium* contains three genomes: 1) a nuclear genome that comprises 14 linear chromosomes;2) a linear mitochondrial genome that is one of the smallest known ; 3) a 35 kb circular plastid genome of red-algal origin, that is housed in the apicoplast.

The life cycle of all species of human malaria parasites is characterized by an exogenous sexual phase (named sporogony), in which multiplication occurs in several species of *Anopheles* mosquitos, and an endogenous asexual phase (named schizogony) which take place in the vertebrate host.^1^ The latter phase begins when sporozoites following the inoculation by mosquito, enter the parenchymal cells of liver, and undergo their development and multiplication, a process that is known as, pre-erythrocytic schizogony. In the vertebrate host the life cycle of *Plasmodium* is characterized by a tissue phase in the liver, followed by a blood phase, that is ultimately responsible of the appearance of clinical manifestations. It is generally believed that once an infectious female anopheline mosquito injects sporozoites in the blood, they are able to travel quickly to the liver where they invade the host’s hepatocytes. However, new findings seems to indicate the existence of a more complex invasion process characterized by the persistence of sporozoites in human dermis for hours with a slow release into capillaries as well as migration through lymphatic system.^3^,^4^ In the liver the sporozoites undergo their replication becoming a tissue schizont that contains thousands of merozoites (about 10,000 in *P.vivax/P.ovale* and up to 30,000 in *P.falciparum*). This exoerythrocytic schizogony is characteristic of each species and takes a minimum maturation time of 6 days in *P.falciparum* and a maximum of 16 days in *P.malariae*. The mature schizont ruptures (together with the infected hepatocytes) releasing merozoites that once in the blood actively invade erythrocytes using an acto-myosin motor system. The tissue phase extinguishes itself at this point with the notable exception of *P.vivax* and *P.ovale* (and certain other primate species such as *P.cynomolgi*) who are able to persist in the liver as “hypnozoites” that are eventually responsible of the phenomenon of relapse. The blood cycle begins when the merozoites invade the erythrocytes and the period of time from the infection by mosquito bite and the first appearance of the trophozoites in the erythrocytes is called “prepatent period” ; this period is characteristic of each species and is constant. The prepatent period lasts 9 days in *P.falciparum*, 11–13 days in *P.vivax*, 10–14 days in *P.ovale*, 15 days in *P.malariae* and 9–12 days in *P.knowlesi* (Table 2). Within the erythrocytes, trophozoites mature over the course of 24–72 hours, a phenomenon that is again species-dependent, with the production of blood-schizonts each one containing 6 to 36 merozoites. The rupture of erythrocytes releases into the blood a new wave of merozoites that are able to infect other erythrocytes. The rupture of the schizonts is followed by the appearance of the malaria febrile paroxysm classically lasting 8 to 12 hours ( “Golgi cycle”) and characterized by three stages : 1) the cold stage marked by the rapid rise of the temperature associated with chills; 2) the hot stage with the temperature peak, skin vasodilatation, headache, myalgias; 3) the sweat stage with defervescence. However, it should be noted that the appearance of typical febrile periodicity (tertian or quartan fever) requires the synchronization of the blood parasite cycles and since symptomatic cases of malaria are usually observed earlier than in the past, this feature is now rarely encountered in the clinical practice in western countries. During the process of schizogony some of the merozoites differentiate into sexual forms- female (macrogametocytes) and -male (microgametocytes), respectively, which are responsible, if ingested by a female anopheline mosquito of the definite sporogonic cycle. It was initially believed that gametocytes persisted in the blood for long periods of time but, a study by Hawking and coworkers, conducted on *P.knowlesi*, *P.cynomolgi* and *P.cathemerium* (a duck *Plasmodium*) contrasted this view.^5^ They showed that gametocytes develop to the stage of infectivity for mosquitoes a few hours longer than their asexual cycle and then remain mature for only a short period of hours (5–12) finally degenerating and disappearing from the blood. The molecular mechanism that triggers the development of gametocytes as well as the factors that determine the sex of gametocytes are poorly understood; however, duration and stages of gametocytogenesis vary among *Plasmodium* species.

The sexual development of malaria parasite (sporogonic cycle) will be completed only when mature female and male gametocytes are ingested by a biologically suitable species of female *Anopheles* mosquito during a blood meal. A mosquito blood meal is, on average, 2 to 3 μL, and should contain at least one male and one female gametocyte to be infective.

Host location by the mosquito is mediated by physical (heat, moisture, visual) and chemical cues that play a role during orientation and landing. 6,7 It is known that skin bacteria play an important role in the production of human body odour and convert non-volatile compounds into volatile compounds with characteristic smells. In a recent elegant experiment Verhulst et al. studied the attractiveness of 48 human males to *Anopheles gambiae* (a nocturnal highly anthropophilic species) one of the major malaria vector in Africa. They showed that individuals with higher skin microbial diversity and higher abundance of *Pseudomonas* and *Variovorax* spp. were less attractive to the mosquitoes and hypothesized an in-built defence system controlled by the Major Histocompatibility Complex genes.^8^

Once in the midgut of the insect the macrogametocyte (*i.e.* female) is released by the erythrocytes to became a macrogamete (within 5 minutes) whereas the male counterpart (microgametocyte) divides its nucleus more slowly (about 20 minutes) into eight flagellated microgametes responsible of the fertilization of macrogamete a process that takes one hour.^9^ The zygote that is formed by the fusion of the two nuclei evolved into a slowly motile ookinete that actively penetrate the peritrophic membrane and the midgut epithelium. Twenty-four to forty-eight hours after the ingestion of blood, the oocyst develops and, subsequently, its single nucleus divides repeatedly until the formation of a mature oocyst that contains thousands of sporozoites. The time span required to development of mature oocyst is quite variable (7–30 days), characteristic of each species and influenced by ambient temperature. Oocyst development is the longest developmental phase as well as the only extracellular of the *Plasmodium* life cycle. The sporozoites actively escape from the oocyst and only 25 % of those liberated by oocyst migrate through the hemocoelomic fluid to the acinal cells of salivary glands, where after about a day of residence, they became highly infective.^10^ They are irreversibly programmed for their journey in the vertebrate host because they completely lost their capacity to re-infect salivary glands.^10^ Once that the mosquito bites the piercing proboscis probes the dermis for a blood vessel and ejects saliva, which, via its anticoagulant activity, facilitates blood ingestion.^11^ Sporozoites present in the salivary ducts are ejected during skin probing in a number generally not exceeding 10–100.

### Plasmodium falciparum

*Plasmodium (Laverania) falciparum* (Welch, 1896) is the highly pathogenic and most deadly parasite causing malaria in humans. It was discovered in 1880 by Charles Alphonse Laveran, a French Army Surgeon, deployed in Constantine (Algeria) and originally named by himself *Oscillaria malariae*.^12^ Examining under a microscope a drop of blood from a young soldier with fever, Laveran observed some spherical and crescent-shaped bodies with actively moving filaments ; he was looking at exflagellation of a male gametocytes of *P.falciparum* a phenomenon that was subsequently explained by Maccallum.^13^ Exflagellation of the microgametocyte in the life cycle of malarial parasites occurs in the stomach of mosquitoes after ingestion of an infected blood meal but in rare cases it can be observed also in the peripheral blood smear of infected humans, generally as the consequence of extended delay in slide preparation or following warming.^14^

Eighty-five countries are actually classified as endemic for *P.falciparum* malaria with 2.57 billion people living in area at risk for transmission of this infection.^15^ Of these, 1.44 billion people live in area of stable transmission, mainly in Africa (52% of the global total) and Central, South and East Asia (46%).^16^

The estimate of the burden of *P.falciparum* clinical malaria is a difficult task complicated by several factors such as inadequate and incomplete national reporting systems and inaccurate diagnoses with possible overestimate disease rates. By using improved cartographic and epidemiological data sources combined with geostatistical space-time joint simulation framework, Hay and coworkers, estimated that in 2007 there were 451 (95 % confidence interval 349–553) million clinical cases of *P.falciparum* malaria.^17^ Sixty percent (271 million) occurred in Africa (including Saudi Arabia and Yemen), and 39 % in the 19 countries of Central and South East (CSE) Asia with the highest-burden countries being Nigeria and Democratic Republic of Congo in Africa and India and Myanmar in CSE Asia region.^16^ These figures are in contrast with the most recent estimates by the WHO that reported in 2010 an estimated 216 million (149–274 million) episodes of malaria worldwide with 655,000 deaths (537,000–907,000).^18^

The whole genome of *P.falciparum* has been sequenced and published in 2002 being a major advance in the struggle against this lethal parasite.^19^ The nuclear genome, distributed in 14 linear chromosomes, consists of about 5,365 genes of whom 1,817 have known functions (Table 3). Of these 1,817 functional genes of *P.falciparum*, 81.6 % have been shown to be conserved with those of *P.vivax* and only 334 genes (18.5%) were unique to the former species.^20^ In comparison with *P.vivax* and *P.knowlesi* genomes, *P.falciparum* shows an higher content of A+T and despite having the smallest genome size it displays the highest content of simple sequence repeats (SSRs) that are supposed to be responsible of genome complexity responsible for rapid evolutionary adaptation (Table 3).^21^ The proteomic characterization of the four stages of the parasite life cycle (sporozoite, merozoite, trophozoite, gametocyte) showed that about half of the sporozoite proteins are unique to this stage whereas trophozoite, merozoite and gametocytes had between 20 % and 30 % unique proteins.^22^ Moreover only 152 proteins (6%) were shared to all four stages and the high degree of proteome diversity of each stage of the *Plasmodium* life cycle suggested a highly coordinated expression of genes involved in common process. However, the comparative genomics analyses of *Plasmodium* genomes indicated the genes mediating parasite-host interactions are frequently restricted to a single *Plasmodium* species. For *P.falciparum*, the *P.falciparum* erythrocyte membrane protein 1 (PfEMP1) that is known to be encoded by about 60 members of the specific *var* gene family, is considered the principal virulence factor of *P.falciparum* because PfEMP1 proteins, expressed on the surface of infected erythrocytes, are able to mediate adhesion to both uninfected and host endothelial cells.^19^

The exoerythrocytic schizogony of *P.falciparum* is a rapid process that is usually completed in five and half days with the production of a schizont, characterized by bizarre forms, containing a very high number of merozoites. Following the rupture of liver schizonts, each released merozoite invade an erythrocyte a complex process that requires a recognition between receptors (on erythrocyte) and ligands (on merozoite). In the case of *P.falciparum*, early studies identified as possible receptors for merozoite invasion sialic acid, glycophorin A, B and C.^23^–^25^ Another sialic acid-independent erythrocyte receptor for *P.falciparum* adhesin PfRh4, the complement receptor 1 (CR1) has been identified in 2010.^26^ However, none of these receptor-ligand pairs were shown to be essential in all parasite strains tested. More recently, Crosnier and colleagues identified *basigin*, an antigen of the Ok blood group, as the receptor essential for the erythrocyte invasion by *P.falciparum*.^27^ Basigin is also known as CD147, EMMPRIN and M6 and is a member of the immunoglobulin superfamily (IgSF) that is implicated in many biological functions. In more detail, basigin proved to be a receptor for the PfRh5 a unique protein among the EBAs and Rhs that cannot be deleted in any *P.falciparum* strain.^27^

Erythrocytic schizogony is characterized by the appearance in the blood of young rings whereas the maturation stages are rarely seen in the peripheral blood. There is no enlargement of the infected erythrocytes during the development and the mature schizont usually contains 8–32 merozoites. The gametocytes of *P.falciparum* develop in the internal organs and do not appear until about eight to ten days after the start of parasitaemia. *P.falciparum* gametocytogenesis has been characterized through five morphologically distinct substages.^28^,^29^ Immature *P.falciparum* gametocytes (stage I–IV) are sequestered away from circulation and only the mature crescent-shaped gametocytes (stage V) are released in peripheral blood where they finally become infectious to mosquitoes.^29^

Until the last year, the closest ancestor of *P.falciparum* was considered to be a chimpanzee parasite, *P.reichenowi*, which was believed to have diverged from its human counterpart 5–7 Myears.^30^–^32^ However, a recent paper, published in 2010, in whom the Authors analyzed more than 2500 samples of ape fecal material as a source of *Plasmodium* DNA, using a single-genome amplification strategy, challenged this view.^33^ The Authors, claimed that human *P.falciparum* sequences constitute a single lineage nested within the G1 clade of gorilla parasites thus suggesting that human *P.falciparum* is of gorilla origin and not of chimpanzee.^33^,^34^ Moreover, several papers published in 2010, showed that *P.falciparum*, once considered strictly human specific, can infect bonobos, chimpanzees and gorillas and thus these African apes might serve also as possible reservoir for the malignant form of human malaria.^35^–^38^ In this regard, five new phylogenetic species within the *Laverania* subgenus have been identified in just one year providing a new representation of the phylogeny with two groups. Group A that includes three species : *P.GorA* (that infects Gorillas), *P.gaboni* and *P.billbrayi* (both infecting chimpanzees) and group B that includes *P.falciparum* and *P.reichenowi* and *P.GorB* (that infects gorillas) and *P.billcollinsi* (that infects chimpanzees).^39^ Finally, researchers from USA, confuted the new view that *P.falciparum* was transferred by gorilla to human and on the basis of their analysis of 45 orthologous gene sequences, indicated that ape infections with *P.falciparum* might be a very recent phenomena.^40^

### Plasmodium vivax

*Plasmodium vivax* (Grassi and Feletti, 1890) is responsible of the so called “benign tertian fever” although the lack of life-threatening complications associated with this infection have been recently questioned by several reports.^41^,^42^ It was identified as a separate malaria parasite together with *P.malariae* in 1886 by Camillo Golgi who described the typical “tertian” and “quartan” fever paroxisms and confuted the Laveran’s postulate of the existence of a single malaria species.^43^ In 1890 Giovan Battista Grassi and Raimondo Feletti named them *Haemamoeba vivax* and *H.malariae*.^44^ The whole genome of *P.vivax* Salvador I strain was sequenced and published in 2008: with its 26.8-megabase (Mb) nuclear genome is higher than that of *P.falciparum* and displays chromosomes that are unique among human *Plasmodium* species with an isochore structure.^45^ It contains 5,433 predicted protein coding genes and represents the most GC-rich (42.3 %) *Plasmodium* genome sequenced to date (Table 3). A recent analysis of proteome of *P.vivax* was able to identify 7 proteins which were entirely specific to *P.vivax* and 16 proteins that did not share any homology in *P.falciparum* (2 *Vir* and 8 *P-fam* proteins) probably implicated in virulence/antigenicity of *P.vivax*.^46^

*Plasmodium vivax* is present throughout the tropics with low rate of infection in western and central sub-Saharan Africa. According to recent estimates 40% of the world’s population (2.6 billion people) is at risk of *P.vivax* transmission with between 130–435 million clinical episodes of vivax malaria each year.^47^,^48^ The origin of *P.vivax* has been, as in the case of *P.falciparum*, a debatable question with different hypotheses; the abundance of simian malaria species observed in Southeast Asia together with morphological and biological characteristics shared by *P.vivax* with macaque parasites where the arguments used to place the origin of *P.vivax* in Southeast Asia.^49^ On the contrary, the high prevalence of Duffy negativity (*i.e*. the lack of the Duffy blood group antigen) among human population throughout sub-Saharan Africa has been indicated to argue an African origin for *P.vivax* 50 Subsequent studies using data regarding complete mitochondrial genomes and nuclear and plastid genes argued that *P.vivax* was introduced into *Homo sapiens* in Asia by a species of *Plasmodium* parasitic to macaques.^51^–^53^

Sporozoites of *P.vivax*, once in the liver, differentiate either into early, primary tissue schizonts or into hypnozoites which are responsible for late relapse of the infection. The term “hypnozoite” was probably coined and adopted for malaria by Markus in 1978 whereas the biological proof of the existence of hypnozoites is the direct consequence of the work by Krotoski and coworkers.^54^–^56^

The biological determinant that supports the active or dormant development pathway is still unknown although the relapsing nature of *P.vivax* malaria was described at the end of nineteen century by Thayer, Bignami and Manson as outlined in the recent elegant review by White.^57^

During the erythrocytic development of *P.vivax* all forms can be found in the peripheral blood and in most stages the appearance is larger that in the other species of human *Plasmodia*. It is also responsible of the enlargement of host cells thus increasing its deformability.^1^ The parasite preferentially invades young red blood cells (*i.e* reticulocytes) an issue that seems to limit its reproductive capacity with level of parasitaemia that rarely exceed 2 % of circulating erythrocytes. The invasion of erythrocytes by *P.vivax* merozoites requires interaction with the Duffy antigen receptor for chemokines (DARC) with Duffy-negative individuals considered to be naturally resistant to this human malaria parasite.^58^ The high proportion of Duffy-negative people in West and Central Africa has long be viewed as the most plausible explanation of the rarity of *P.vivax* malaria in those geographical areas. However, in recent years several investigators either in Africa and South America were able to demonstrate that *P.vivax* has evolved and adapted in a way that circumvent this pathway and it is able to invade erythrocytes also in individuals who lack the Duffy antigen on their red cells.^59^–^61^ The young throphozoite grows rapidly and exibits the characteristic malaria pigment; subsequently it assumes an amoeboid activity and a large vacuole forms a “hole” within the ring until the division of the nucleus begins. The mature schizont contains on average 12 to 18 merozoites and fills the entire host cell.^1^,^49^

Gametocytes production probably start with the first generation of merozoites of *P.vivax* and can be detected within 3 days after the first asexual parasites are observed.^29^

One of the most intriguing and debatable issue regarding *P.vivax* is whether or not it exists as a single species or on the contrary different species or subspecies are represented.^62^–^64^ Li and coworkers showed that *P.vivax* comprises two distinct lineages with phenotypic difference with respect to their preferred mosquito vector and with a distinct chromosomal translocation.^65^ On the basis of differences on the incubation period and on relapse intervals it has been known for a long that different phenotypes (*P.vivax* Chesson, *P.vivax* Madagascar, St.Elizabeth, *P.vivax* North Korean, *P.vivax* Hibernans) are responsible of different presentation.^62^,^63^ Madagascar and St.Elizabeth strains (considered the typical *P.vivax*) usually cause a primary illness two weeks after mosquito inoculation with subsequent relapse after an interval of 7–10 months but with shortened subsequent inter-relapse intervals. On the contrary the *P.vivax hibernans* (distributed in northern Europe and Russia) presented a primary infection 8–10 months after inoculation. Finally, the *P.vivax* Chesson strain shows very frequent relapses and high relapse rate. The exact trigger for the activation of hypnozoites is not understood and various theories such external stresses, seasonal stimuli or mosquito bites, have been proposed.^57^,^64^

In the *Anopheles* mosquito, after fertilization the sexual cycle takes 8–10 days at 28 °C and 16 days at 20 °C whereas below 15 °C the completion of the sporogonic cycle is unlikely.^1^

### Plasmodium ovale

*Plasmodium ovale* (Stevens, 1922) was discovered in 1922 by Stephens who observed it in the blood of an East african patient with malaria erythrocytes with oval shape and fimbriated edges and named the parasite *P.ovale*.^66^ Using the sequences of the small subunit ribosomal RNA (SSUrRNA) gene it has been established that *P.ovale* belong to 2 genetic haplotypes named classic and variant.^67^ Both the classical and variant types are morphologically indistinguishable and occurred in sympatry worldwide ; based on the observation that no evidence of inter-or intragenic recombination could be observed among samples coming from different part of the world, Sutherland and coworkers raised the possibility of the existence of 2 species and proposed to name these species *P.ovale curtisi* (classic type) and *P.ovale wallikeri* (variant type) in honour of 2 malariologists : Christofer Curtis (1939–2008) and David Walliker (1940–2007).^68^,^69^ *P.ovale* is distributed in sub-Saharan Africa, South-east Asia (Philippines, Myanmar, Vietnam, Thailand), Middle East, the Indian subcontinent, Papua New Guinea and Irian Jaya and East Timor in Indonesia ; it has never been reported from South America.^70^ It has been estimated that the global burden of *P.ovale* in Africa might exceeds 15 million cases annually.^68^ The prepatent period (*i.e.* the interval between sporozoites inoculation and the first detection of parasites in peripheral blood) of *P.ovale* is between 12 and 20 days, with a median of 14,5 days.^71^ Parasitaemia is usually low during *P.ovale* infection probably as the consequence of restricted development in younger erythrocytes; in a study regarding 90 patients the maximum parasite levels ranged between 380 and 27,660/μL with a mean maximum parasite level of 6,944/μL.^71^ The changes produced by *P.ovale* on the infected erythrocytes are similar to those seen in *P.vivax* whereas the schizonts and gametocytes may resemble those of *P.malariae*.^1^ The completion of the sporogonic cycle in the mosquito takes 12–14 days at 28 °C.

*P.ovale* is generally regarded as responsible of a relapsing infection originating from dormant (“hypnozoites”) exoerythrocytic stages in the liver.^1^,^71^ According to Collins and Jeffery, the only demonstration of the existence of liver hypnozoites in human was that of Garnham and coworkers who made a liver biopsy in a volunteer that was deliberately fed by *Anopheles* mosquitoes infected with a Liberian strain of *P.ovale*.^71^,^72^ However, in a recent provocative article by Richter and colleagues, the Authors reported the rarity of relapses in naturally acquired infections and hypothesized that some of these cases might be due to *P.vivax* misidentified as *P.ovale*.^73^ Moreover, they claimed that in *P.ovale* infection, hypnozoites have never been demonstrated by biological experiments but in their paper failed to cite the work by Garnham.^72^,^73^ After treatment of a primary attack the relapse interval for *P.ovale* has been described to be in the range of 17 to 255 days;^71^ however, delayed primary attacks have been observed after as long as 4 years and in a recent paper of mostly PCR confirmed cases of *P.ovale*, a primary attack was observed after 53 months.^74^,^76^

Recently in a study conducted in Cameroon, Duval and coworkers found for the first time two chimpanzees infected by human *P.ovale* a condition that raise the possibility that cross-species exchange might be more important than previously thought with the potential role of African great apes as reservoir for human malaria parasites.^77^

### Plasmodium malariae

*Plasmodium malariae* (Laveran, 1880), responsible of the “quartan malaria”, is present worldwide in all major malaria-endemic regions but with a scattered distribution.^70^,^78^ Infections caused by *P.malariae* are most common in sub-Saharan Africa and southwest Pacific and less frequently encountered in Asia, Middle East, Central and South America. As for *P.vivax* also for *P.malariae* the relationship between the life cycle of development (respectively 48- and 72-hours) and the periodicity of the fever paroxysm were elegantly explained by Camillo Golgi in 1886 although the two parasites were identified as separate species by Grassi and Feletti.^43^,^44^

The parasite is characterized by a slow development either in the *Anopheles* mosquito (15 days) and in human (15 days in the liver, 72 hours in the blood). *P.malariae* is considered to be the precursor of *P.brasilianum* a parasite that infects New World monkeys and has naturally adapted to it;^1^,^78^ both *Plasmodia* are able to infect either humans and monkeys. *P.malariae* is responsible of low grade parasitaemia, rarely exceeding 30,000 parasites per microliter, probably as a consequence of the low number of merozoites produced per erythrocytic cycle together with the 72-hour developmental cycle and the preference to infect older erythrocytes. The pre-patent period for *P.malariae* is extremely variable with a range of 16 to 59 days. No quiescent liver stage forms have been identified for *P.malariae* but this parasite is able to persist in the blood with low level parasitaemia for extremely long periods and perhaps for the entire life of the human host causing recrudescence even after more than 30–40 years or longer.^1^,^78^–^80^ Moreover, chronic *P.malariae* infection was linked to nephrotic syndrome in Nigerian children about 50 years ago, and it is believed to be caused by immune complex deposition on the basement membrane.^81^,^82^ The young trophozoites are similar to those of *P.vivax* (although their cytoplasm is thicker and they stain more deeply); a characteristic feature of *P.malariae* is the appearance of band form with the trophozoite that stretch across the entire width of the cell. The mature schizont has an average of 8 merozoites that are sometimes arranged symmetrically around the centre with a daisy appearance.^1^ The sporogonic cycle in the *Anopheles* mosquito takes 30–35 days at 20°C but may be as short as 14 days at 28°C.

### Plasmodium knowlesi

*Plasmodium knowlesi* (Sinton and Mulligan 1932) is a simian plasmodium that was probably first described by the Italian malariologist Giuseppe Franchini in the blood of *Macaca fascicularis*. 83,84 Subsequently it was studied by Napier, Campbell, Das Gupta and Knowles and finally was completely characterized by Sinton and Mulligan who named it *P.knowlesi* in honour and acknowledging the original work of Dr Knowles.^85^–^87^ *P.knowlesi* was employed in the treatment of general paresis of insane (*i.e.* neurosyphilis) until 1955 when it was finally abandoned due to the increased virulence of the organisms after multiple passages in humans.^85^,^88^ The first natural infection of humans was serendipity observed in an American Army patient who was deployed in Peninsular Malaysia and developed malaria on the way home.^89^ The availability of molecular diagnostic tools recently helped to distinguish *P.knowlesi* from *P.malariae* and to identify it as an important cause of human malaria not only in the Peninsular Malaysian Borneo but also in other parts of South-east Asia.^2^,^90^ Phylogenically, *P.knowlesi* is more closely related with *P.vivax* than other humans *Plasmodia* and in a similar way the process of merozoites invasion of erythrocytes requires the interaction of Duffy-bindings proteins (DBP) with the Duffy antigen receptor for chemokines (DARC).^1^,^91^,^92^ However, important phenotypic differences with *P.vivax* exists such as absence of a dormant liver stage, host blood cell preference and length of asexual cycle. The genome of *P.knowlesi* has been sequenced and described showing important differences with those of *P.falciparum* and *P.vivax* (Table 3).^93^ *P.knowlesi* was the first malaria parasite in which antigenic variation was demonstrated to occur.^94^

Long-tailed (*Macaca fascicularis*) and pig-tailed macaques (*M.nemestrina*) are the main natural hosts of *P.knowlesi*.^85^ After infection, all the developmental stages of the malaria parasite life-cycle are observed in the peripheral blood. The intra-erythrocitic life-cycle is of 24 hours (unique for all malaria parasite of primates) with an asynchronous development and it is not restricted to young or old cells.^85^,^89^ The young ring forms appear very similar to those of *P.falciparum* whereas during subsequent stage of maturation the intra-erythrocyte parasites resemble the band forms observed in *P.malariae* infection.^2^,^85^ The mature schizont contains as many as 16 merozoites with an average of 10. The sexual forms grow more slowly than asexual forms and take usually 48 hours to complete their development; the macrogametocyte at the end of maturation is spherical with a blue-stained cytoplasm and fills the host cell whereas the microgametocyte is sometimes smaller and with a pink-stained cytoplasm. High level of parasitemia and severe manifestations resembling those of *P.falciparum* malaria have been described for *P.knowlesi* with a possible lethal outcome.^94^,^95^

## Conclusions

Our knowledge of the biology of human malaria parasites has dramatically improved in the last few years thanks to the whole sequencing of DNA of the two most important human *Plasmodia: P.falciparum* and *P.vivax*. Moreover, the introduction of molecular techniques has greatly improved the identification of malaria parasites at the species level. In this regard, the availability of species-specific polymerase chain reaction allowed Balbir Singh and his coworkers to recognize the significant role of *P.knowlesi*, a natural plasmodia of macaques, as a cause of human malaria in the Southeast Asia. This discovery, together with the possibility to use non-invasive methods to supply animal DNA, renewed the interest to study malaria parasite of apes increasing the membership clade of *P.falciparum* and *P.ovale* and finally showing that *P.falciparum* is diverse and not human-host restricted.

## Figures and Tables

**Table 1:** Classification of human protozoa of the genus P*lasmodium*

**Domain**	Eukaryota
**Kingdom**	Chromalveolata
**Superphylum**	Alveolata
**Phylum**	Apicomplexa
**Class**	Aconoidasida
**Order**	Haemosporida
**Sub-order**	Haemosporidiidea
**Family**	*Plasmodiidae*
**Genus**	*Plasmodia*
**Sub-genus**	*Plasmodium; Laverania*
**Species**	*P.falciparum* *P.malariae* *P.ovale* *P.vivax* P.knowlesi*

* *P.knowlesi* is a primary parasite of monkey that can infect humans

**Table 2:** Characteristics of infection with the five species of Plasmodia infecting humans

Characteristics	*Plasmodium falciparum*	*P.knowlesi*	*P.malariae*	*P.ovale*	*P.vivax*
Pre-erythrocytic stage (days)	5–7	8–9	14–16	9	6–8
Pre-patent period (days)	9–10	9–12	15–16	10–14	11–13
Erythrocytic cycle (days)	48	24	72	50	48
Red cells affected	All	All	Mature erythrocytes	Reticulocytes	Reticulocytes
Parasitaemia per μL					
• Average	20,000–500,000	600–10,000	6000	9000	20,000
•Maximum	2,000,000	236,000	20,000	30,000	100,000
Febrile paroxysm (hours)	16–36 or longer	8–12	8–10	8–12	8–12
Severe malaria	Yes	Yes	No	No	Yes
Relapses from liver forms	No	No	No	Yes	Yes
Recurrences	Yes (treatment failure)	Yes	Yes (as long as 30–50 years after primary attack)	No	Yes (treatment failure)

**Table 3:** Comparative genome size, whole genome SSR, A+T and G+C content of the three sequenced genome of *Plasmodium* species causing human malaria

*Plasmodium* species	Overall genome size (Mb)	Number of genes*	Overall SSR content (%)	Overall A+T content (%)	Overall G+C content (%)
*Plasmodium vivax*	26.8	5,433	3.8	58	42.3
*Plasmodium knowlesi*	23.5	5,188	4.92	63	37.5
*Plasmodium falciparum*	23.3	5,403	10.5	81	19.4

* Including pseudogenes and partial genes, excluding non-coding RNA genes; SSR=simple sequence repeats; A=adenine; T=thymine; G=guanine; C=cytosine
